# Unique presentation of T-cell/histiocyte-rich large B cell lymphoma complicated with hemophagocytic lymphohistiocytosis: Case report and review of the literature

**DOI:** 10.5937/jomb0-48290

**Published:** 2024-06-15

**Authors:** Andrej Pešić, Vojin Vuković, Sofija Kozarac, Vladimir Otašević, Tamara Bibić, Biljana Mihaljević, Darko Antić

**Affiliations:** 1 University Clinical Center of Serbia, Clinic for Hematology, Belgrade; 2 University of Belgrade, Faculty of Medicine, Belgrade

**Keywords:** hemophagocytic lymphohistiocytosis, T-cell/histiocyte-rich large B cell lymphoma, refractory lymphoma, immune-related adverse event, case report, review of the literature, hemofagocitna limfohistiocitoza, T-cell/histiocyte-rich difuzni B-krupnoćelijski limfom, refraktarni limfom, imunološki posredovana komplikacija, prikaz slučaja, pregled literature

## Abstract

Hemophagocytic lymphohistiocytosis (HLH) is a rare, lifethreatening hyperinflammatory disorder characterized by dysfunction of NK cells and cytotoxic lymphocytes. We present a rare case of a patient diagnosed with HLH who presented with persistent fever during treatment for refractory T-cell/histiocyte-rich large B-cell lymphoma (TCHRLBCL), highlighting the challenges of managing HLH in the context of refractory lymphoma. According to our review of the literature, this is the first case of HLH that developed several months into treatment for refractory TCHRLBCL and not in close temporal relation to lymphoma diagnosis.

## Abstract (sr)

Hemofagocitna limfohistiocitoza (HLH) je redak, životno ugrožavajući poremećaj koji se karakteriše disfunkcijom NK ćelija i citotoksičnih limfocita. Prikazujemo redak slučaj pacijenta sa dijagnozom HLH sa tegobama u vidu perzistentne temperature u toku lečenja refraktarnog "T-cell/histiocyte-rich" difuznog B-ćelijskog limfoma (TCHRBCL), izdvajajući izazove u lečenju ovakvog profila pacijenta. Uz osvrt na naš pregled literature, ovo je prvi opisan slučaj HLH koji se javio nekoliko meseci nakon početka lečenja TCHRBCL, a ne u uskoj vremenskoj korelaciji sa dijagnozom limfoma.

## Introduction

Hemophagocytic lymphohistiocytosis (HLH) is a rare, life-threatening hyperinflammatory condition characterized by dysfunction of lymphocytes, leading to cytokine-mediated tissue damage and multiple organ dysfunction [1]. The mechanisms underlying HLH are not completely understood. The main pathological feature seen in almost all cases of HLH is a defect in cytotoxic T cells and natural killer (NK) cells, leading to a loss of cytotoxic cell-mediated regulation and subsequent uncontrolled inflammation [2]. It is postulated that CD8+ T cells and NK cells are unable to kill target cells due to defective cytolysis, which leads to sustained cell-cell contact and antigen presentation. These defective cells also have reduced susceptibility to activation-induced apoptosis in addition to their inability to kill antigen-presenting cells. Sustained activation of CD8+ T cells results in the release of large amounts of IFN-γ, a potent activator of macrophages. In response, macrophages produce high levels of IL-1, IL-6, and TNF-α, which may account for many clinical features of HLH, such as fever, pancytopenia, splenomegaly, hyperferritinemia and hypertriglyceridemia [1] [2].

HLH is traditionally classified into familiar and acquired (secondary) form, based on the presence or absence of the mutations involving key genes in the NK and CD8+ cell-mediated cellular cytotoxicity [3]. Acquired HLH occurs in association with infections, malignancies (mostly hematologic), and rheumatologic diseases [2]. Considering the high mortality rate, delayed diagnosis due to low disease prevalence and a variety of nonspecific symptoms, this condition remains a major clinical challenge [3] [4]. Malignancy-associated HLH (M-HLH) usually presents concomitantly with the presentation of malignancy or rarely precedes its diagnosis [5] [6]. The pathogenesis of M-HLH is not entirely understood. One possible explanation for its development is unregulated production of IFN-γ and TNF-α by transformed cells, which drives HLH and markedly lowers the threshold for disease [1]. It should be noted that most cases of M-HLH are associated with hematological malignancies, with a majority [7] [8], of them linked to T or NK cell lymphoma [9], implicating a common pathophysiological trait seen in all of HLH: CD8+ and NK cell dysfunction.

We present a unique case of a patient developing HLH during the treatment of refractory T-cell/ histiocyte-rich large B cell lymphoma (TCHRB-CL), another rare entity that accounts for less than 5% of all cases of diffuse large B-cell lymphoma (DLBCL) [7].

### Case description

**Figure 1:**
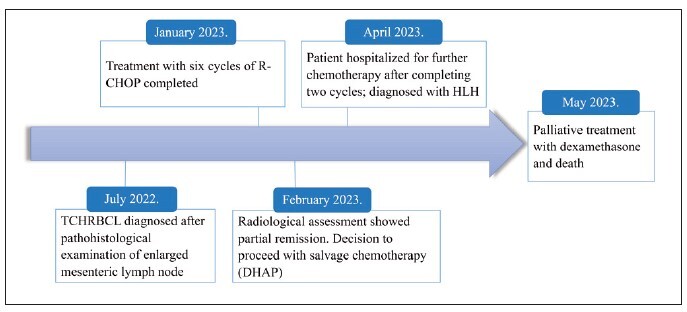
Timeline from the diagnosis of TCHRLBCL to the development of HLH and subsequent death. DHAP: Dexamethasone, Cytarabine, Cisplatin, R-CHOP: Rituximab, Cyclophosphamide, Doxorubicin, Vincristine (Oncovine), and Prednisone

A 32-year-old Caucasian male was hospitalized for a newly diagnosed TCHRBCL. The diagnosis was obtained after the biopsy of an enlarged mesenteric lymph node. His medical history was significant for intellectual disability with no known underlying cause and hypertension. He had prominent facial features, including low-set ears, prognathism and premature hair hypopigmentation that appeared at the age of fourteen. His family history was unremarkable. The patient was subsequently classified as IV clinical stage according to the Ann Arbor classification. The response to the first line of treatment was insufficient as the patient achieved only partial remission. Before the third cycle of salvage chemotherapy, the patient presented to our ward with a persistent fever lasting for two weeks, for which he was hospitalized at the local health center. Blood culture, urine culture, nose and throat swabs, chest X-ray, stool culture and testing for *Cl. difficile* did not show any signs suggestive of infection and no pathogenic microorganisms were isolated. He did not respond to broad-spectrum antimicrobial therapy, including meropenem, vancomycin, fluconazole, and acyclovir, and he was consequently transferred to our facility. On admission, significant laboratory findings showed pancytopenia, low fibrinogen, high total bilirubin, elevated AST and ALT, elevated LDH and grossly elevated ferritin. We suspected that the patient developed HLH. Further examination revealed splenomegaly and hepatomegaly on ultrasound, as well as elevated levels of soluble CD25 and hypertriglyceridemia. Myelogram showed no evidence of hemophagocytosis and NK cell activity assay was not performed due to technical limitations. Considering the presence of fever, pancytopenia, hyperferritinemia, hypertriglyceridemia, hypofibrinogenemia, splenomegaly and elevated soluble CD25, the diagnosis of hemophagocytic lymphohistiocytosis in the background of TCHRLBCL was made, based on the revised 2004 Histocyte Society diagnostic criteria [8]. Table 1 summarizes the initial laboratory findings when TCHRLBCL was diagnosed and findings when HLH developed. *(this is the approximate position of the table in the text)*. NGS (next generation sequencing) TruSightOne panel was performed to rule out late-onset familiar HLH but the results were negative. Because patient’s condition deteriorated while ongoing salvage chemotherapy and given that etoposide, a drug commonly used in the treatment of HLH, was contraindicated due to marked hyperbilirubinemia, monotherapy with corticosteroids was started. It was essentially a form of palliative treatment, and the patient eventually died. Figure 1 summarizes the clinical course chronologically.

**Table 1:** Summary of laboratory findings during the initial hospitalization, before the second cycle of salvage chemotherapy and when the diagnosis of HLH was made. WBC-white blood cells, AST-aspartate transaminase, ALT-alanine transaminase, LDH-lactate dehydrogenase, CRP-C-Reactive Protein

Laboratory <br>findings	Reference range	Initial admission <br>(August 2022)	Second cycle of salvage <br>chemotherapy (March 2023)	Development of HLH <br>(April 2023)
Hemoglobin, g/L	130-180	104	110	71
WBC count, x10^9^/L	3.6-10	3.5	3.5	2.1
Neutrophil count, <br>x10^9^/L	2-7.5	2.61	1.2	1.5
Platelets, x10^9^/L	150-450	148	102	31
AST, U/L	0-37	32	52	295
ALT, U/L	0-41	21	41	153
Total bilirubin, μmol/L	0-20.5	7.1	8.2	38.4
LDH, U/L	0-248	369	548	3493
Ferritin, μg/L	30-400	1295	1180	21258
Fibrinogen, g/L	2.2-5.5	6.4	6.4	1.2
Triglyceride, mmol/L	0.0-1.7	1.63	1.48	4.59
Creatinine, μmol/L	70-115	228	110	47
β2 microglobulin, mg/L	1.21-2.70	19.01	6.42	7.96
CRP, mg/L	0-5.0	56.1	39.9	58.1
H-score and estimated <br>probability of HLH		81; <1%	60; <1%	286; >99%

## Discussion

As previously mentioned, M-HLH and malignancy usually present together. This was not the case with our patient, as he developed HLH nine months after the initial diagnosis. As previously mentioned, association with lymphoma is well established, particularly with NK and T-cell lymphoma [9]. HLH is less often associated with B-cell lymphoma [10], and association with TCHRLBCL is extremely rare.

Diagnosis of HLH per HLH-2004 criteria requires five of the following eight features: fever, splenomegaly, cytopenias affecting 2 of 3 cell lineages (hemoglobin <90 g/L, platelets <100 x 10^9^/L and neutrophils <1 x 10^9^/L), hypertriglyceridemia and/or hypofibrinogenemia, hemophagocytosis, hyperferritinemia, high soluble CD25 levels and low/absent NK cell activity [8]. Our patient fulfilled six criteria. As previously mentioned, NK cell activity was not performed due to technical limitations. Additionally, no evidence of hemophagocytosis, the most striking feature of the disease, was found. The prevalence of hemophagocytosis in association with HLH ranges from 25 to 100% [11]. Furthermore, as per HLH-2004 criteria, the evidence of hemophagocytosis is not mandatory for the diagnosis of HLH, and it should never be made or excluded solely on the presence or absence of hemophagocytosis [12]. It is also interesting to note that tissue hemophagocytosis is frequently observed in the absence of HLH; with infections, blood transfusions, autoimmune disease, and bone marrow failure [1]. Another useful instrument that aided our diagnosis was the H-score, which was developed to facilitate and expedite the diagnosis of HLH in adults [13]. The main difference from the HLH-2004 criteria is that it considers underlying immunodeficiency as a diagnostic criterion and lacks criteria such as soluble CD25 levels and NK cell activity, two tests that are not readily available in many institutions. According to the H-score, our patient had a >99% probability of HLH based solely on the clinical picture and routine laboratory parameters, again implicating its greatest advantage.

It remains unclear whether our patient had a genetic predisposition for HLH. Distinctive facial features and neurocognitive impairment suggest a possible genetic disorder. With the increasing availability of genetic testing, it became apparent that the first significant episode of familial HLH can occur from prenatal presentations to the seventh decade of life [2]. One study reported that 14% of the 175 adult patients with HLH had disease-associated mutations, reported cases of late-onset familial HLH first manifesting as lymphoproliferative disease [14] [15] [6]. Chediak-Higashi syndrome (CHS), Griscelli syndrome type 2 (GS2), and Hermansky-Pudlak syndrome type 2, immune deficiency disorders characterized by lysosomal dysfunction are also associated with HLH [2]. Our patient did not have a history of frequent infections, but it is interesting to note that he had grayish hair since childhood, a feature common in these syndromes [16]. This was the rationale for performing NGS. The results were compared to reference humane genome (hg19). Unfortunately, no pathogenic, potentially pathogenic or variants of un known significance associated with HLH were found. Since NGS does not perform as well at detecting structural rearrangements or copy number variations (CNVs), perhaps the result was false negative given that our patient had a probability of the above-mentioned genetic abnormalities due to his undetermined developmental disorder [17].

To the best of our knowledge, eight cases of HLH secondary to TCHRLBCL were described in the English literature [18] [19] [20] [21] [22] [23] [24] [25]. Their summary is displayed in Table 2. It is interesting to note that all of the patients presented in advanced clinical stages, with only one patient classified as stage III and all of the others as stage IV according to Ann Arbor classification. Eight of them were male, with only one female patient. The majority presented in the fourth decade, as did our patient. No other patient had a history of intellectual disability or suspicion of a hereditary disorder, and we were the only one who performed NGS in this small cohort of patients. Most patients achieved clinical remission (CR) of lymphoma and HLH. However, longer follow-up data for the outcome of the reported cases are lacking, with the longest CR reported at 24 months [24].

**Table 2:** Features of previously described cases of HLH associated with TCHRBCL ASCT: autologous stem cell transplant; CR: clinical remission, CS: corticosteroids, DA-R-EPOCH: dose adjusted Rituximab, Etoposide, Prednisone, Vincristine (Oncovin), Cyclophosphamide, Adriamycin (Doxorubicin); DHAP: Dexamethasone, Cytarabine, Cisplatin; MOPP-ABV: mechlorethamine, vincristine, procarbazine, prednisone/doxorubicin bleomycin, and vincristine; R-CHP: Rituximab, Cyclophosphamide, Doxorubicin, Prednisone; R-CHOP: Rituximab, Cyclophosphamide, Doxorubicin, Vincristine (Oncovin), and Prednisone; R-COPADM: Rituximab, Cyclophosphamide, Oncovin (Vincristine), Prednisone, Adriamycin (Doxorubicin), Methotrexate; R-CYM: Rituximab, Cytarabine, Methotrexate

Case <br>(reference)	Sex	Age	Clinical stage <br>(Ann-Arbor)	Diagnosis of <br>HLH relative <br>to the diagnosis <br>of TCHRBCL	Treatment	Outcome
Devitt et al. <br>[20]	Male	30	Stage IV <br>(retroperitoneal nodes, <br>bone marrow, lung)	Concurrent	Dexamethasone and <br>etoposide, DA-R-EPOCH	CR
Metts et al. <br>[22]	Male	16	Stage IV <br>(retroperitoneal nodes, <br>bone marrow, spleen, <br>liver)	Two weeks <br>before	Dexamethasone, <br>Cyclophosphamide <br>and Rituximab, R-CHP, <br>R-COPADM, R-CYM	CR
Mahtat et al. <br>[19]	Male	52	Stage III <br>(Axillary, inguinal nodes)	Concurrent	CS, R-CHOP	CR, relapse after <br>ten months
Mehta et al. <br>[23]	Male	20	Stage IV <br>(diffuse lymph nodes, <br>bone marrow, liver)	Concurrent	CS, R-CHOP	CR
Mitterer et al. <br>[24]	Female	30	Stage IV <br>(diffuse lymph nodes, <br>liver)	One month <br>before	MOPP-ABV; <br>methotrexate, vincristine <br>andetoposide <br>followed by ASCT	CR
Ojo et al. <br>[18]	Male	56	Stage IV <br>(diffuse lymph nodes, <br>bone marrow, seminal <br>vesicles, CNS)	Concurrent	Dexamethasone, <br>R-EPOCH with <br>intrathecal <br>methotrexate	Unknown
Ibrahim et al. <br>[21]	Male	43	Stage IV <br>(retroperitoneal, <br>inguinal nodes, <br>liver, spleen)	Concurrent	Dexamethasone, <br>R-CHOP	CR
Aljitawi et al. <br>[25]	Male	34	Stage IV <br>(bone marrow)	At lymphoma <br>relapse	Unknown	Death
Our case	Male	32	Stage IV <br>(retroperitoneal nodes, <br>bone marrow, sacrum)	9 months after	R-CHOP, DHAP, <brr>Dexamethasone	Death

The most striking difference we noted with our case was the time association between TCHRLBCL and HLH. Seven of the previously described cases had a diagnosis of HLH presenting concurrently with or one month before the diagnosis of lymphoma. Only one case described a patient who presented as HLH at the time of lymphoma relapse and it was the only case where death was reported [25], implicating the significance of time association between lymphoma presentation and HLH on prognosis and clinical outcome. Concurrent lymphoma and HLH presentation provides the opportunity to target both diseases using first-line anti-lymphoma treatment, which is the best strategy for resolving M-HLH. Unfortunately, this strategy could not be applied in our case due to the refractoriness of lymphoma, and this had a significant impact on the patient’s outcome.

We faced the dilemma of determining the exact moment HLH developed. Per HLH-2004 criteria, our patient did not fulfill enough of them for the diagnosis on his first admission, nor he did while being hospitalized for the second cycle of salvage chemotherapy. Looking at the Table 1, which summarizes the patients’ laboratory findings, it can be concluded that earlier, unnoticed development of HLH was highly unlikely. Eight months into lymphoma treatment, patient did not meet the criteria for fever, cytopenias affecting two or more lineages, or hypofibrinogenemia/ hypertriglyceridemia. Only splenomegaly and hyperferritinemia were present, both of which are frequently observed in patients with lymphoma. Further workup for HLH (soluble CD25 levels, bone marrow aspiration, NK cell activity) wasn’t performed in that period due to low clinical suspicion. Table 1 also contains patient’s H-score, which, as previously mentioned, facilitates the diagnosis of HLH. It provides additional valuable evidence in determining when HLH actually developed, with a probability >99% in the period when the diagnosis was made and a probability of less than 1% only one month prior to the diagnosis. It should be noted that hemophagocytosis on bone marrow aspirate wasn’t included in the H-score since this procedure wasn’t performed in the respective period due to low clinical suspicion. Even if the patient had evidence of hemophagocytosis at the time of lymphoma diagnosis and eight months into the treatment, the H-score would still estimate the probability of HLH at around 1%. Additionally, as previously mentioned, the patient didn’t have evidence of hemophagocytosis even when the diagnosis of HLH was actually made.

In summary, diagnostic criteria were not met until nine months after starting treatment for lymphoma. It should be noted that cases of smoldering adult HLH before acceleration to a full-blown disease were previously reported [26]. Perhaps the combined effect of malignancy and worsening immunosuppression due to cumulative immunochemotherapy ultimately triggered HLH.

Treatment of M-HLH is directed at both the malignancy and hyperinflammation. The patient’s lymphoma was refractory with HLH further complicating clinical course. TCHRLBCL was also previously noted to have a worse prognosis than DLBCL, NOS (not otherwise specified), with a five-year survival with R-CHOP reported at 46% [27]. Additionally, mortality in HLH is high: in a cohort of 260 patients the ICU mortality rate was nearly 60% [4]; another study reported all patients with malignancy-associated HLH (M-HLH) died within a median time of 22 days (range 0–108) following diagnosis of HLH [28]. Underlying lymphoma, low platelet count, elevated AST and LDH, all of which were present in our patient, were previously linked with early death related to HLH [29]. Additionally, generally accepted guidelines for managing M-HLH are lacking [30]. Our patient had a poor prognostic profile with very limited treatment options. His clinical status deteriorated rapidly with worsening hepatic and renal function precluding the use of further intense regimens. We considered the original HLH-94 protocol consisting of etoposide and dexamethasone. Since marked hyperbilirubinemia contraindicated etoposide administration, we were limited to dexamethasone monotherapy, which was essentially a form of palliative treatment.

This case emphasizes the need for new therapeutic options in M-HLH. Clinical studies are lacking due to low disease prevalence and its acuity. Emapalumab, an IFN-g-blocking antibody, was approved by FDA in 2018, but only for the treatment of young children with HLH [1]. Ruxolitinib, a Janus kinase 1/2 inhibitor, is another exciting drug offering inhibition of multiple cytokine pathways overstimulated in HLH. Studies combining ruxolitinib with other drugs in the treatment of HLH give promising results [31] [32] [33]. Since ruxolitinib, in contrast to etoposide, isn’t contraindicated in patients with hepatic impairment, perhaps the combined therapy of ruxolitinib and dexamethasone would provide a better outcome for our patient.

HLH is a rare entity with many non-specific signs and symptoms overlapping with lymphoma which leads to the delay of diagnosis. The presence of persistent fever with biochemical parameters of high cell turnover in a patient with refractory lymphoma doesn’t necessarily indicate only lymphoma progression, but perhaps another immunological complication developing. To our knowledge, HLH occurring several months after TCHRLBCL has not been previously reported. Alertness is mandatory to make the correct diagnosis. We indicate the challenges in managing HLH in the context of refractory lymphoma, as treatment options are very limited. New, more specific and less toxic therapy is of vital importance for this rare group of patients.

## Dodatak

### Funding

This work was supported by grant No. III 41004, Ministry of Education, Science and Technological Development, Republic of Serbia.

### Acknowledgments

Authors acknowledge and thank all of the clinical staff, nurses, laboratory technicians and other medical doctors from the Clinic for hematology, University Clinical Center of Serbia who were engaged in the management of this patient.

### Data Availability Statement

The original contributions presented in the study are included in the article/supplementary material, further inquiries can be directed to the corresponding author/s.

### Conflict of interest statement

All the authors declare that they have no conflict of interest in this work.

### List of abbreviations

HLH, Hemophagocytic lymphohistiocytosis; <br>TCHRLBCL, T-cell/histiocyte-rich large B-cell lymphoma; <br>M-HLH, Malignancy-associated HLH;<br>DLBCL, NOS, Diffuse large B-cell lymphoma, not otherwise specified
